# It’s hard to reach the “hard-to-reach”: the challenges of recruiting people who do not access preventative healthcare services into interview studies

**DOI:** 10.1080/17482631.2018.1479582

**Published:** 2018-06-18

**Authors:** Lauren Rockliffe, Amanda J. Chorley, Laura A. V. Marlow, Alice S. Forster

**Affiliations:** Research Department of Behavioural Science and Health, UCL, London, UK

**Keywords:** Qualitative research, prevention, acceptability of healthcare, interview, recruitment activities, refusal to participate

## Abstract

In this article, we discuss the challenges faced in recruiting “hard-to-reach” groups for interview studies, specifically those who do not access preventative healthcare services. We do this by reflecting on the varying success of different recruitment methods we have used in two recent studies; one investigating ethnic disparities in human papillomavirus vaccination uptake and another exploring difference in cervical screening non-participation. Engaging new community groups to help with recruitment proved particularly difficult, as did recruiting online. Our most successful recruitment methods included recruiting through community groups with whom we had previously established relationships, recruiting through schools and re-contacting participants who previously completed a related survey. We conclude that successful recruitment is dependent on study awareness and engagement. We urge others to be transparent in reporting recruitment methods in order to benefit the qualitative research community and suggest that details are published as supplementary material alongside qualitative articles in future.

## Introduction

In the UK, various preventative healthcare services are offered within the National Health Service (NHS) such as cervical screening, childhood immunisation and smoking cessation services, all of which are provided free at the point of delivery. The utilisation of healthcare services such as these can reduce mortality and the incidence of illnesses (Sabates & Feinstein, 2006) as well as potentially reducing overall healthcare costs for the NHS (Labeit, Peinemann, & Baker, 2013). In addition, preventative healthcare provides individuals with knowledge and a sense of security about their own health and the health of their loved ones (Sabates & Feinstein, 2006). These services are offered to members of the public who meet certain criteria (e.g., based on age or gender). However, some groups within the general population are less likely than others to access the preventative healthcare services offered to them.

Groups of the population that are difficult to reach or involve in research or public health programmes are typically referred to as “hard-to-reach” groups and may include those living in faith based communities, migrants, newly arrived residents or those living in vulnerable social and/or economic situations (Shaghaghi, Bhopal, & Sheikh, 2011). However, the “hard-to-reach” label has been applied to many different groups in many different contexts. For the purpose of this paper, we define “hard-to-reach” as groups of the population who do not access preventative healthcare services.

Individuals who fall within this “hard-to-reach” category do not form a homogenous group, with specific sub-groups who are less likely to access each individual service varying widely. For example, women between the ages of 25 and 29, lesbians, sex workers and those from non-White British backgrounds are less likely to attend cervical screening (Fish & Anthony, 2005; Health and Social Care Information Centre, 2015; Jeal & Salisbury, 2004; Moser, Patnick, & Beral, 2009) and individuals under the age of 65, those who smoke and those from Black African and Chinese backgrounds are less likely to attend NHS Health Checks for the prevention of cardiovascular disease (Robson et al., 2016).

Despite having some idea about the types of people that make up “hard-to-reach” groups, we are still unclear about what can be done to engage with and to encourage these groups to access preventative healthcare services. Theories from behavioural science have enabled us to understand some of the factors that may affect participation generally, such as individuals’ attitudes, perceived barriers, perceived risks and social norms (Ajzen, 1991; Rosenstock, 1966). Furthermore, The Preventative Health Model (PHM; Myers et al., 1994) posits a range of factors that may affect decision making about taking preventative action, such as background factors (i.e., demographic characteristics, medical history, past health behaviour), representation factors (i.e., severity of health condition, susceptibility, curability, worry, salience and coherence), social influence factors (i.e., the clinician-patient relationship, social norms, health locus of control) and program factors (i.e., promotional communications). Based on this framework, Myers et al. (1994) suggest that the extent to which an individual views apreventative health behaviour, such as screening for example, as effective, convenient, beneficial and important, will be positively related to their intention to take action and directly to the action itself.

Despite having some idea about potential contributing factors, we know relatively little about the specific reasons for non-participation as these groups are notoriously hard to recruit into health research. The sub-groups of the population who fail to access preventative healthcare services are unlikely to be motivated to participate in research about such services. This creates a paradox whereby health researchers are unable to engage “hard-to-reach” groups to find out why they are not accessing certain healthcare services and what can be done to facilitate the utilisation of them. In other words, it’s hard to reach the “hard-to-reach”.

Members of “hard-to-reach” groups tend to have poorer health outcomes than those who utilise preventative healthcare services (Hewitson, Glasziou, Watson, Towler, & Irwig, 2008; Landy, Pesola, Castanon, & Sasieni, 2016; Weedon-Fekjær, Romundstad, & Vatten, 2014). These groups would therefore benefit the most from interventions to increase uptake of the services available to them. This is one of the primary reasons it is so important for researchers to access and engage with these groups, to find out why they do not access certain healthcare services, and whether non-participation is due to an informed choice or is due to potentially modifiable barriers preventing access.

There are likely to be a variety of reasons why individuals do not access preventative healthcare services; for some individuals prioritsing day-to-day tasks or having a busy lifestyle may prevent them from fully engaging with the services offered (e.g., Chapple, Ziebland, Hewitson, & McPherson, 2008; Chorley, Marlow, Forster, Haddrell, & Waller, 2016; Forster et al., 2016a), whereas others may make a deliberate decision to not access the services. Of course, for some individuals the decision to not access these services will be the right one for them. However, to make such a decision, people must be provided with relevant, unbiased information about the potential consequences of making their choice, and the choice must be autonomous and free from coercion (Jepson, Hewison, Thompson, & Weller, 2005).

When undertaking research with “hard-to-reach” groups, the aims are often to understand people’s experiences of accessing healthcare services and/or to identify barriers preventing them from doing so. Interviews are a widely used method for such research and allow access to the private, often contradictory and complex beliefs people hold (Pope & Mays, 1995). Interviews provide the opportunity to obtain more detail about an issue or experience and can uncover concerns that had not previously been anticipated or considered (Pope, Van Royen, & Baker, 2002). Elicitation of such information is essential in identifying perceived barriers to accessing preventative healthcare services. However, recruiting participants into interview studies, whether they are part of a “hard-to-reach” group or not, is typically a difficult task. Interviews can be time consuming, they may compromise privacy and can be both intellectually and emotionally demanding (McCracken, 1988).

Despite the difficulties, numerous qualitative studies have managed to successfully recruit “hard-to-reach” groups (e.g., Chorley et al., 2016; Forster et al., 2016b; Hall et al., 2016; Ingall & Cropley, 2010; Palmer, Thomas, von Wagner, & Raine, 2014) and these provide us with an insight into the different types of recruitment methods that have been employed. A number of studies have described identifying non-attendeers from health records held by service providers and contacting the individuals directly by phone or letter to invite them to participate (Hall et al., 2015; McCaffery et al., 2001; Oscarsson, Mijma, & Benzein, 2008). Other studies have described forging links with community leaders to access their networks and approach individuals directly (Quaife, Marlow, McEwen, Janes, & Wardle, 2016) or have offered financial incentives to encourage participation (Ellis et al., 2015).

Researchers can also learn from the approaches that are commonly used to recruit other groups that are “hard-to-reach”, aside from those who do not access preventive healthcare services. Respondent-driven sampling (RDS), for example, is an advanced type of chain-referral sampling which involves providing incentives for those participating in the study, as well as those recruiting other eligible participants (Heckathorn, 1997). This is a recruitment method that has been used to access groups of the population such as drug users (e.g., Abdul-Quader et al., 2006), men who have sex with men (MSM; e.g., Hladik et al., 2012), and female sex workers (e.g., Johnston, Sabin, Hien, & Huong, 2006), all of which have been described as “hard-to-reach” groups in other contexts. While RDS is more often used to recruit for survey studies, the approach has also been successfully used to recruit “hard-to-reach” groups, such as female sexual minorities and drug users, into interview studies (e.g., Martin, Johnson, & Hughes, 2015; Witteveen, Van Ameijden, & Schippers, 2006). Alternative approaches commonly used to recruit other “hard-to-reach” populations include time-location (or time-space) sampling, and venue-based sampling, where recruitment is conducted at times and locations within the community where groups of “hard-to-reach” populations are likely to be found (Karon, 2005) (e.g., Mutagoma et al., 2017; Wittenberg et al., 2015), indigenous field worker sampling, where individuals from the local community are trained to recruit and collect data in place of researchers (Shaghaghi et al., 2011) (e.g., Greene et al., 2009) and community-based outreach (e.g., Halcón & Lifson, 2004). Examples of successful recruitment into interview studies using such approaches are provided by Hunt, Moloney, and Fazio (2011) who used venue-based sampling, amongst other approaches, to recruit participants into an in-depth interview study about their drug use and youth culture, and by Elliott, Watson, and Harries (2002), who used peer interviewers to recruit and interview parents who use illegal drugs.

Whilst there are many methodological papers discussing sampling techniques used to recruit other “hard-to-reach” groups, such as those previously mentioned, the same cannot be said for our predefined “hard-to-reach” group: people who do not access preventative healthcare services. Papers discussing recruitment strategies for this group are far and few between and more notably, little information is provided within non-methodological papers to describe the challenges associated with recruitment; how easy or hard the researchers found the recruitment process, whether there were any alternative failed recruitment attempts or what challenges had to be overcome to recruit a sufficient number of participants. For example, Wong, Wong, Low, Khoo, and Shuib (2009) conducted an interview study with Malaysian women who had never attended cervical screening. The article reports that women were recruited using purposive and snowballing recruitment methods, however it is not clear how many women were approached, how long the recruitment process took or if there were any unexpected barriers to recruitment. A further example is that of Ellis et al. (2015) who describe the recruitment of individuals who did not take up an invitation to a NHS Health Check. The article reports that forty-one interviews were conducted but states that low response rates meant they had to contact additional patients; further reflection on this issue would be useful for other researchers wanting to replicate this approach. Similarly, McCaffery et al. (2001) describe recruiting individuals uninterested in attending bowel cancer screening. Potential participants were contacted by telephone and a sample of sixty were recruited. However, for future research it would have been useful to know the total number of individuals contacted or how long the recruitment process took, for example.

A gap appears to exist in the literature whereby articles fail to explicitly address the difficulties associated with recruiting “hard-to-reach” groups specifically when conducting interview studies, and fail to provide any meaningful reflections on the recruitment process. With this article, we aim to go some way towards bridging this gap by discussing the challenges we have faced when recruiting those who do not access preventative healthcare services into interview studies. We intend to provide a useful summary of the methods that we have and have not found effective for the recruitment process.

The following examples provide details of two studies we conducted in 2015 and 2016 respectively, focusing on the utilsation of preventative healthcare services in the UK. The first is an interview study which sought to understand reasons why parents from ethnic minority backgrounds are less likely to consent to human papillomavirus (HPV) vaccination for their daughters. The second is an interview study investigating why some groups of women do not participate in cervical screening or do not intend to in the future.

## Recruitment examples

### Context

Cervical cancer is a disease affecting thousands of women in the UK each year (Office for National Statistics, 2015). In recent decades considerable advances have been made in reducing the incidence of the disease through a population-based screening programme (Peto, Gilham, Fletcher, & Matthews, 2004). Further reductions in incidence are expected in coming decades following the introduction of the HPV vaccination programme in 2008. However, there are concerns about falling cervical screening levels (Health and Social Care Information Centre, 2015) and the potential exacerbation of health inequalities due to differences in HPV vaccination coverage between ethnic groups (Fisher, Audrey, Mytton, Hinkman, & Trotter, 2014).

### The research team

The following two studies were carried out by a team of researchers working at University College London (UCL), in the Research Department of Behavioural Science and Health (previously the Health Behaviour Research Centre). The core team working on these studies included five researchers; two Research Assistants, two Senior Research Associates and a Principal Research Associate. All researchers are White British females who, at the time of conducting the studies, were aged between 29 and 42.

### Study one

Study one was a qualitative interview study looking to understand ethnic disparities in uptake of the HPV vaccination. Parents of adolescent girls, aged between 14 and 16 years of age (in school years 9 to 11), were invited to participate. This included parents from Black and Asian Minority Ethnic (BAME) backgrounds whose daughters had and *had not* received the vaccination, as well as parents from White British backgrounds whose daughters had not received the vaccination. This eligibility criteria was set to ensure we recruited parents of adolescent girls who had already been offered the HPV vaccination (The HPV vaccination is recommended for girls aged 12–13 years old (in school year 8)). Non-vaccinating White British participants were recruited as a comparison group. As the purpose of this article is to discuss “hard-to-reach” groups, only the recruitment of non-vaccinating parents will be discussed (The HPV vaccine is currently administered in two doses (prior to September 2014 it was administered in three doses). Non-vaccinating parents are defined as any parent whose daughter had either not started or not completed the vaccine series).

Patient privacy regulations in the UK mean that, unless in exceptional circumstances, patients must consent to their medical records and contact details being used for purposes beyond their healthcare. We deemed that this “hard-to-reach” group were unlikely to provide consent to contact prior to being invited to participate, which would result in a small and engaged population from which to sample from. We therefore used five different approaches to facilitate recruitment; recruitment through secondary schools, through community groups, online advertisements, “snowballing” and via personal outreach. Recruitment took place over 12 months and a total of 22 non-vaccinating parents were recruited.

### Study two

Study two refers to an ongoing study which aims to create a typology of cervical screening non-participants, using both quantitative and qualitative methods. Women aged between 25 and 64 (i.e., all women of screening eligible age), who are not up-to-date with cervical screening recommendations, are eligible to participate. The first stage of this study involved conducting a survey with the aim of categorising respondents as one of five types of non-participants (1. unaware of screening; 2. unengaged; 3. undecided; 4. decided not to attend; 5. decided to attend but did not act on it) according to the Precaution Adoption Process Model (PAPM; Weinstein, 1988). Women were recruited on our behalf by a market research company who employed a random location sampling method (random location sampling involved randomly sampling from over 100 predefined geographic areas within Great Britain). Based on the findings from this survey, we set out to recruit 40 women spread across each of the identified non-participant groups, to take part in interviews. We particularly wanted to focus on recruiting those women who had never heard of cervical screening, and women who had heard of screening but had never attended, as we had previously identified an absence of their viewpoints in the published literature (Chorley et al., 2016).

Recruitment began in June 2016 and is ongoing. We initially intended to recruit all interview participants from the pool of non-participating women who had completed the initial survey and consented to be contacted for future research projects. However, a lack of success with this method led to us trying a further three approaches; “snowballing”, advertising on social media, and through community groups. To date, 13 women have been recruited over six months.

Both studies, and any subsequent amendments, received ethical approval from the University College London Research Ethics Committee (study one: 3758/001; study two: 7585/002).

To ensure that the recruitment materials developed for use in study one were appropriate for use with our target populations, the materials were reviewed by a lay group of parents with whom we conducted several user involvement groups. Group members provided feedback on the materials and subsequent changes were made. The recruitment materials used in both studies are available in Supplementary Material.

Table I provides a summary of the recruitment approaches used in both studies.

**Table I.** Summary of approaches used and number of participants recruited.

Details	Study one	Study two
Participants recruited (N)	22	13
Sample	Parents whose daughters had not received the HPV vaccination	Women who had never heard of cervical screening or who had heard of it but never attended
Secondary schools	458 schools contacted; 27 assisted with recruitment; 10,282 parents invited to participate; 17 participants recruited	-
Survey	-	345 participants completed survey; 163 women invited to participate; offered £20 incentive; 16 responded; 9 participants recruited
Community groups	195 groups contacted; 11 assisted with recruitment; no participants recruited	1 group contacted and assisted with recruitment (previous link with group); 3 participants recruited
Online advertising	Adverts posted on 3 websites for max. 2 months; no participants recruited	Adverts posted on 3 websites for min. 1.5 months; 6 women left contact details, 1 participant recruited
*Facebook advertising*	Advert posted for 15 days; seen by 30,145 users; clicked on by 233 users; 1 participant recruited	Advert posted for 7 days; offered £50 prize draw + £20 incentive; seen by 15,558 users; clicked on by 176 users; no participants recruited
Snowballing and personal outreach	3 participants recruited via snowballing; 1 participant recruited via personal outreach	No participants recruited

### Secondary schools

For study one we contacted 458 London schools by letter and/or email and asked if they would allow us to send recruitment letters to parents of female students in years 9 to 11. All secondary schools based in Greater London, excluding all boys’ schools, were eligible to be contacted. Of those approached we received a response from 97 schools (21%) and 27 (6%) agreed to help. We then contacted the parents of 10,282 students by letter or email and as a result, recruited 17 non-vaccinating parents for the study (77% of the 22 participants recruited).

The main challenges we faced with recruiting through schools were primarily at the school level (i.e., schools not wanting to take part, administrative issues). While we did successfully recruit 17 non-vaccinating parents this way, this represents just 0.2% of the parents who were contacted, suggesting significant barriers to participation, as more than 17 non-vaccinating parents would be expected in such a large sample. It is difficult to know why we did not have a greater response rate, although this may be due to the paradox mentioned previously, where people who do not access preventative healthcare services are less likely to be engaged with research about preventative healthcare, and less related to the recruitment method used.

### Survey

As part of study two, 345 survey respondents who were categorised as non-participants in cervical screening gave their consent to be re-contacted. We initially believed that using this list as a recruitment pool would provide the following two advantages; firstly, the women’s survey responses allowed us to identify which of the five non-participant categories they fell into. This was considered a particular advantage as screening behaviour is not an easily identifiable characteristic, and adverts for studies about cervical screening are unlikely to interest women who are unaware or unengaged with screening. Secondly, we believed that because these women had already completed the survey and had consented to be contacted for future research, they would likely be somewhat engaged with the research process and therefore more likely to participate in the interview study than comparable women in the general population.

From this list of non-participants we invited a total of 163 women to take part in interviews. The first round of invitations were initially sent to 75 of these women offering no incentive, however we received no response. Following this, we modified our recruitment strategy to include an offer of a £20 Boots voucher as an incentive to take part (Boots is a store that sells pharmacy, health and beauty products). The decision to do so was a personal decision made by all members of the research team. Although there is some controversy regarding the use of financial incentives within research, we do not believe the incentive offered created any ethical issues in relation to women feeling obliged to participate out of financial need, given the voucher format and the relatively low value of the incentive (For a more detailed discussion of ethical considerations when using financial incentives in qualitative research see Head (2009)). All 75 women who received the initial invitation were re-contacted with this offer and the remaining 88 women were sent the modified invitation letter. Following the offer of an incentive we received 16 contact forms, and to date nine women have been interviewed (69% of the total recruited).

### Community groups

Both studies attempted to recruit participants through community groups. We wanted to recruit individuals from BAME backgrounds and as community groups are often defined by characteristics such as ethnicity this seemed like a sensible approach. For study one it was important to recruit people from BAME backgrounds as we were interested in ethnic disparities in uptake of the HPV vaccination and for study two the results of the survey had identified that BAME women, and those who had English as a second language, were more likely to be unaware of cervical screening than other groups.

For study one we contacted 195 community groups via email and 42 were also sent information in the post. We initially approached groups working with adolescent girls or parents but to increase the reach of our materials we also translated the information into Somali and Bengali and approached groups working with those communities (but not necessarily with parents or adolescent girls). These languages were selected as they are spoken as a main language by significant sized populations in London (Gopal & Matras, 2016; Krausova & Vargas-Silva, 2013). We received a response from 24 of the groups contacted (12%) and of those, 11 groups (6%) assisted with recruitment either by promoting our study to their members, or by allowing us to attend meetings and talk to members directly (we attended five community groups and spoke to approximately 60 members). However, we recruited no non-vaccinating parents from community groups.

For study two, we contacted one community group with whom we had previously worked, via email, asking if they were able to identify any women who fit the study criteria. The community group leader approached women on our behalf and arranged interviews for us with those who agreed to participate. Thus far we have recruited three women from this one community group (23% of the total recruited).

Despite our best efforts to contact relevant community groups for study one, the demographic makeup of the groups meant that often the members were ineligible to participate (something we only found out after making contact). In some cases we accepted that it would be a fruitless task to give presentations to such groups, for example where the members were too young to have daughters of the right age for vaccination. However, in certain cases we were happy to give presentations to members in the hope that they might know of others who were eligible, for example where members were too old to have daughters of the right age, but might have granddaughters of an appropriate age (and therefore could put us in contact with potentially eligible parents). Regardless of this, some group leaders believed that their members would not be interested in the study and were not willing for us to attend.

Another challenge we faced in study one was that many of the groups comprised members who did not speak English as their first language. This often resulted in group leaders rejecting our request to visit the group, although some were willing if we were accompanied by an interpreter. We were able to arrange for Bengali and Somali interpreters (two commonly spoken languages) but due to financial constraints it was not possible to arrange for someone to interpret less commonly spoken languages, which limited the groups we were able to visit.

A number of group leaders also requested payment in exchange for their assistance (e.g., for contacting group members who they thought might be eligible), whilst others believed that their members would not be interested unless we could provide them with a monetary incentive. Again, due to financial constraints, this was not something we could offer.

### Online advertising

Towards the end of the recruitment period for study one we decided to advertise the study online. Modified versions of the same advert were posted on Mumsnet (http://www.mumsnet.com/), Netmums (http://www.netmums.com/) and Call for Participants (https://www.callforparticipants.com/) for a maximum of two months. The adverts provided concise study information in 160 words or less and provided a link to an online version of our participant information sheet, as well as contact details of the lead researcher. The adverts posted on these websites generated very little interest with only two individuals expressing an interest in participating, neither of whom did.

An advert for study two was also posted on Mumsnet, Netmums and Gransnet (https://www.gransnet.com/) for a minimum of one and half months (currently ongoing). This was again a pragmatic decision made by the research team and based on the assumption that online advertising may increase the reach of our recruitment materials. The advert was a short 128 word post, providing details about the study, researcher contact details and linking to a survey we created using SurveyMonkey (https://www.surveymonkey.co.uk/). The survey asked women questions about cervical screening and other preventative health behaviours. If women indicated that they did not go for screening, or did not plan to in the future, they were directed to a page inviting them to take part in the interview study and were asked to provide their contact details if interested. In addition to the £20 voucher offered for participating in the interview study, women were also offered a chance to win £50 by completing the survey. A total of 34 women completed the survey and six of these women left their details to be contacted for an interview. Thus far, one woman has participated, two women were found to be ineligible and we have not yet been able to contact the remaining three.

We also advertised on Facebook (https://www.facebook.com/) for both study one and study two. The advert for study one linked to our study website and targeted individuals fitting our eligibility criteria. It ran for 15 days and was seen by 30,145 Facebook users. A total of 233 users clicked through to the study website via the advert, however only one non-vaccinating parent was recruited. The advertisement for study two also linked to the survey, as the previous advertisements did. It ran for one week (correct at time of writing) and reached 15,558 women aged 25 to 34. A total of 176 users clicked through to the survey, but only two went on to complete it, neither of whom provided contact details for the interview study.

The associated costs of online advertising were relatively inexpensive. Two of the websites used (Netmums and Call for Participants) did not charge anything to post an advert. Mumsnet charged a one-off fee of £30.00 (around €34.00 or $USD37.00) and Facebook charged an average of £0.03 for study one, each time someone clicked on the advert (around €0.04 or $USD0.04), and an average of £0.20 (around €0.24 or $USD0.24) for study two.

### Snowballing and personal outreach

Snowballing, also known as chain-referral sampling (a rudimentary version of RDS), was an approach we employed in both studies. For each interview we conducted we asked participants if they knew of anyone eligible who might be willing to take part. Although the majority of participants claimed not to, we recruited three non-vaccinating parents this way for study one, but no participants have been recruited using this method for study two so far.

Personal outreach was used in study one to increase recruitment of participants from ethnic minority backgrounds. We worked with a Somali speaking researcher and a Bengali speaking researcher, both of whom were asked to disseminate information about the study within their communities and to people they knew personally. This approach was similar to that of indigenous field worker sampling, as previously discussed. This method was quite resource intensive for these researchers as they were required to visit people they knew, places of worship and schools, for example, and resulted in the recruitment of only one non-vaccinating parent.

## Discussion

The purpose of this article was to provide an overview of the recruitment methods we employed to recruit “hard-to-reach” groups, in this case those who do not access preventative healthcare services, into interview studies. Drawing on our own experiences we have presented details of the recruitment process for an interview study with parents from ethnic minority backgrounds who had not consented to HPV vaccination for their daughters, and for an interview study with women who do not participate in cervical screening. The intention was to provide detail and transparency about the processes, highlighting the challenges we faced as well as the methods we found most and least effective.

The main challenges faced in study one related to recruiting through community groups. From the outset, it was extremely hard to engage community group leaders and to convince them of the relevance of the study for their members. Of those who were interested, many requested payment for the group, or its members, or required interpreters for us to speak to them. As previously reported, no non-vaccinating parents were recruited using this method. However, this method of recruitment was one of the more successful in study two, due to the low resources required and overall numbers recruited. Almost a quarter of participants were recruited using this method, which involved working with only one community group and required no additional costs or time commitment. We acknowledge however, that the success of this method was due to employing a previously established relationship with a community group leader. It is unlikely that we would have had the same success if approaching unfamiliar community groups as study one demonstrated, and it is likely that far greater efforts would have been required.

The success achieved by utilsing a previously established relationship clearly highlights the importance of forging relationships and connections within the community to facilitate recruitment. Working collaboratively with members of the community not only during the recruitment period, but throughout the entire research process, is an approach used in Community-Based Participatory Research (CBPR), which has generally been shown to raise participation rates and increase the value of studies for both researchers and the community (Viswanathan et al., 2004). CBPR, an overarching term for a variety of approaches such as action research, participatory action research, mutual inquiry and feminist participatory research (Minkler & Wallerstein, 2008), involves working *with* communities, instead of *in* them, and attempts to strengthen the problem-solving capacity of communities through collective engagement in the research process (Viswanathan et al., 2004). This approach acts not as a community outreach strategy but as a systematic effort to incorporate community participation and decision-making into the research process (Wallerstein & Duran, 2006). CBPR is an approach that ensures that research topics reflect local concerns, that brings together partners with various skill sets and expertise, and that enhances the quality and validity of the research by benefiting from the local knowledge of the participants (Israel et al., 2005). Furthermore, CBPR increases the likelihood of overcoming any distrust felt by communities who are commonly the focus of research (e.g., “hard-to-reach” groups) (Israel et al., 2005).

Although we did not employ a CBPR approach in study two, we experienced much greater recruitment success just by establishing a relationship with a stakeholder within the community, than in study one where we did not have this opportunity. This lends support to the CBPR approach and provides a strong argument for using this methodology in future studies with individuals who do not access preventive healthcare services, especially as this is an approach that has been identified as a promising methodology for research aimed at reducing health inequalities (Israel et al., 2005; Wallerstein & Duran, 2006).

Another factor that may have contributed to successful recruitment in study two is the population we were hoping to access. It may be the case that cervical screening is a topic that more people are interested in or is relevant to more people than HPV vaccination. Furthermore, a large number of the community groups contacted for study one were working with BAME communities, which may have impacted on the way in which the study was received. As HPV is sexually transmitted, the topic may be seen as “taboo” or sensitive (Marlow, 2011) and therefore inappropriate to discuss. Furthermore, previous studies have suggested that distrust of scientific investigators and of academic institutions may also act as a barrier to participation for people from BAME backgrounds (Yancy, Ortega, & Kumanyika, 2006).

Heckathorn (1997) found that in the context of respondent-driven sampling the social characteristics of individuals recruiting participants affected the characteristics of those recruited. In particular, the majority of participants recruited often shared similar ethnic backgrounds to those who recruited them. This effect has been shown to dissipate if multiple waves of recruitment are conducted, and an equilibrium in the mix of participants recruited will eventually be achieved (Heckathorn, 1997, 2002). However, this may be an effective approach to use if carrying out single-wave recruitment. Whilst it may not be easy to apply such a method to recruiting groups on the basis of their health participation behaviours alone (e.g., vaccination refusal or screening non-participation), which are not characteristics necessarily obvious to or shared with others, ensuring that face-to-face recruitment of individuals from BAME backgrounds is conducted by a researcher from a similar ethnic background may increase the likelihood of recruiting participants from such backgrounds, who also do not access preventive healthcare services. As previously described, we used a similar approach in study one when a Somali speaking researcher and Bengali speaking researcher were employed to promote the study within their respective communities and to personal contacts.

Despite the difficulties we faced in study one, recruiting through community groups allows for more targeted and efficient recruitment, because groups are often defined by particular characteristics such as gender, age, interests, or ethnic background. For this reason it lends itself to this approach by enabling relatively easy access to individuals with certain characteristics of interest.

In terms of numbers recruited, the most successful method we used in study one was recruiting through secondary schools. The majority of study participants were recruited using this method. Although we did not feel as though we faced any challenges that were specifically related to recruiting a “hard-to-reach” group by using this method, there were challenges nonetheless. The most significant was the high number of schools who did not respond or who did not want to participate. Ultimately the task of engaging schools became a “numbers game”, trying to contact more and more schools until one would agree to take part. Contacting such a large number of schools was hugely time consuming and in hindsight it would have been beneficial to have planned for a longer recruitment phase. An additional reflection is that schools rarely responded to the initial letters we sent out. Making initial contact via email, instead of letter, and following up with a phone call would likely have reduced the amount of time and resources spent, and would be our preferred method of contact in future. It has been suggested that efforts to encourage schools to participate can be facilitated by someone known to the school system (Lamb, Puskar, & Tusaie-Mumford, 2001), as we experienced when trying to engage community groups. It may therefore be beneficial to make use of any previously established relationships that other team members have with schools and to use them as a way to make contact and to introduce the research. In addition, we found that the engagement of key staff members at participating schools facilitated recruitment enormously and that having a key person to liaise with was extremely beneficial and streamlined the process. The concept of engagement and forging relationships within the school context is similar to what has previously been discussed in relation to recruiting through community groups and emphasises the need for greater collaboration and engagement with gatekeepers and stakeholders, regardless of what context they are based.

The most successful method of recruitment in study two was contacting women who were identified as non-participants after taking part in the initial survey. The majority of women were recruited in this way and so in terms of absolute numbers this method was successful. However, offering a monetary incentive meant that this approach required a larger recruitment budget. Researchers may also wish to consider the cost of the initial survey, although in this instance it would have been run irrespective of the interview study, so no additional costs were accrued. The use of incentives are generally acknowledged to be effective at increasing recruitment rates (Singer & Ye, 2012) and this has also been shown to be true for those less likely to take part in research (Guyll, Spoth, & Redmond, 2003). The use of incentives may therefore be a particularly good approach to employ when recruiting “hard-to-reach” groups, and is an approach which should be factored into the budgeting of a study from the outset. However, the ethical implications of using such an approach must be considered, as incentives can be viewed as a form of bribery or coercion (Marteau, Ashcroft, & Oliver, 2009).

It is important to note that the two most successful methods, recruiting through schools and via a survey, involved contacting the largest numbers of people directly (10,282 and 163, respectively). As previously mentioned, recruiting through schools involved contacting as many people as we were able until the recruitment target had been met and perhaps this is ultimately the key to successful recruitment, regardless of what method is used.

That said, online recruitment was not successful in either study and does not appear to follow the same pattern as for school and survey-based recruitment. Despite the extraordinary reach of the Facebook adverts when compared with other methods of recruitment, they were not successful in engaging people with either study, even when the opportunity to be entered into a prize draw was offered, an approach that is often efficacious at increasing survey response rates (Singer & Ye, 2012). A number of factors could have resulted in this outcome; as with the community groups it may have been the topic under investigation that did not engage users or it may have been the visual appeal of the adverts themselves or the way in which the studies were described (see Supplementary Material for recruitment materials used).

Despite our lack of success recruiting online, the potential advantages of advertising via social media should not be overlooked. The reach of social media is enormous; for example sites such as Facebook have an average of 1.18 billion daily active users worldwide (Facebook Investor Relations, 2016). Combined with the ability to target adverts to specific audiences social media sites provide a unique platform from which to recruit participants, and one which may prove more effective for other studies.

The PAPM (Weinstein, 1988), as used in study two, is a process model designed to explain health behaviour. However, this model can be applied to other behaviours, such as research participation and may be able to help us understand how to enhance recruitment. The model consists of seven stages which individuals must progress through in order to make a decision to carry out a behaviour (1. unaware of issue; 2. unengaged with issue; 3. undecided about acting; 4. decided not to act, or 5. decided to act; 6. acting; 7. maintenance). Stages one, two and three are most relevant to the recruitment process and provide us with some indication of how our less successful approaches could be improved. For example, advertising online made people aware of our studies although they chose not to engage with them, suggesting that more could be done to increase the visual appeal and content of such adverts, or to offer greater incentives to participate. Recruiting through community groups only made people aware of the studies when we were allowed access to the groups and of those people who were aware, most were unengaged. This suggests that greater efforts need to be made to engage the gatekeepers of such groups, and their members, something which may be achieved through the use of a CBPR approach, as previously discussed.

The use of a process model, such as the PAPM, provides us with a framework to identify stages in the recruitment process that could potentially be improved. However, other behavioural theories that can be applied within this context provide us with more of an understanding about the mechanisms underpinning the decision-making process. For example, Self-Determination Theory (SDT; Ryan & Deci, 2000) distinguishes between different types of motivation, two of which, extrinsic and intrinsic motivation, may be of particular relevance. While extrinsic motivation refers to carrying out a behaviour or activity in order to attain a separable outcome, intrinsic motivation refers to carrying out a behaviour or activity purely for the inherent satisfaction of doing so (Ryan & Deci, 2000). In the context of research participation, this theory may help to explain people’s motivation for taking part; those who are extrinsically motivated may be more driven by the offer of financial incentives, for example, where as those who are intrinsically motivated may be more driven to participate if the topic under investigation is of particular interest or of personal relevance to them. Conversely, this may help to explain some of the recruitment difficulties we have faced.

Despite discussing our methods in the context of recruiting “hard-to-reach” groups, in this case non-vaccinating parents and women who do not attend cervical screening, these methods are not specific to the recruitment of these groups and may prove successful when recruiting other types of participants. Due to the nature of these “hard-to-reach” groups they comprise only a small population, as the majority of people do access the preventative healthcare services available to them. This alone means that they will inevitably be harder to reach, compared to other more defined “hard-to-reach” groups such as homeless people or drug addicts, for example. Furthermore, those who do not access preventative healthcare services are unlikely to be engaged with research about these services, again making them less accessible.

Both studies had restricted budgets so we did not have funds to pay community groups to assist with recruitment, to pay for translation, or to advertise online for long periods of time. Involving communities far earlier in the research process, for example when applying for grants and designing the research, would enable better planning and budgeting and may have overcome some of these recruitment challenges.

Our examples provide an insight into the challenges associated with using these particular methods to recruit these particular “hard-to-reach” groups. Hopefully by highlighting some of the issues we faced during the recruitment process, others can take these into account when planning their own recruitment strategies and adapt them as appropriate.

The main conclusions we have drawn from our experience is that successful recruitment is dependent on a variety of factors, but study awareness and engagement are vital. Many of the challenges faced were as a result of unawareness or disinterest in the study, both of which could be improved by enhancing recruitment materials, promoting them more widely, and better engaging with communities and stakeholders throughout the research process. A larger recruitment budget would also help to overcome the additional financial issues that affected recruitment. However, the success of any given recruitment method is likely to depend on the characteristics of the participant group, for example younger groups may be more likely to respond to online advertising, whereas face-to-face recruitment may be more effective with older groups. Furthermore, the topic under investigation may have a substantial impact on recruitment efforts, depending on what people find interesting or of personal relevance to them. Ultimately, multi-channel recruitment may be the best approach when recruiting “hard-to-reach” groups to ensure the best response rates.

Being transparent about the recruitment process is something we urge all researchers to do; so often published articles provide limited detail about the approaches taken to recruitment and rarely discuss the challenges they faced. Those conducting research with “hard-to-reach” groups therefore continue to use recruitment methods that have proven to be unsuccessful for others, with no way of knowing otherwise. Whilst reporting guidelines for qualitative research exist, such as the Consolidated Criteria for Reporting Qualitative Research (COREQ; Tong, Sainsbury, & Craig, 2007) and the Standards for Reporting Qualitative Research (SRQR; O’Brian, Harris, Beckman, Reed, & Cook, 2014), and go a long way in helping to ensure the accurate reporting of recruitment processes, no recommendations are made for researchers to produce reflective accounts of their experience, or to document the challenges they faced during the process. If researchers share their knowledge and experience of the methods they have used, the qualitative research community could benefit from not only more successful recruitment, but also from a reduction in the amount of time and resources spent. Going forward, we suggest that additional details of recruitment methods, including recruitment challenges and reflections on the recruitment process, are published as supplementary material alongside qualitative articles in an attempt to provide transparency and in the interest of aiding others working in the field.

## Supplementary Material

Supplemental Material
