# Supplementary material for: It’s hard to reach the “hard-to-reach”: the challenges of recruiting people who do not access preventative healthcare services into interview studies
Source: Int J Qual Stud Health Well-being. 2018 Jun 18;13(1):1479582. doi: 10.1080/17482631.2018.1479582 (PMC6127841; doi:10.1080/17482631.2018.1479582)
Supplement: Supplemental Material [file ZQHW_A_1479582_SM5958.pdf]

The EMPATHIC Study

An invitation to take part in our interview study.

[image]

Summary

Our team is doing research to find out why some girls do get the HPV vaccine and why some girls do not. We would like to speak to parents whose daughters have been offered the HPV vaccine in the past, so we can learn about how they decided whether to let their daughter have the vaccine or not.

We are writing to you to ask if you would take part in this study. If you agree we would come to speak to you about the HPV vaccine. **This would happen at a time and place that is suitable for you.**

The study is being done by researchers from [institution]. Please contact [name] if you would like to talk to her about the study.

Before you decide you need to understand why the study is being done and what it would mean for you. Please read the following information. Talk to your friends and family about the study if you wish.

Please complete the reply slip at the end of this form and send it back in the pre-paid envelope provided if you would like to take part.

[name]
[department], [institution]
[telephone number]

Contents

1 What is HPV?
2 Why is this study being done?
3 Why have I been asked to take part?
4 Do I have to take part?
5 What will happen to me if I agree to take part?
6 What are the possible risks of me taking part?
7 What are the possible benefits of me taking part?
8 Will anyone else know that I have agreed to take part?
9 What will happen if I do not want to continue with the study?
10 What if there is a problem?
11 What will happen to the results of the study?
12 Who is doing the research and who is paying for it?
13 Contact details

1 What is HPV?

Human papillomavirus (also known as HPV) affects the skin and moist areas that line the body. It is spread by skin to skin contact. HPV is common and most people will get infected in their life. In most people HPV causes no symptoms and goes away on its own, but for some people the virus can cause cell changes which can increase the risk of some cancers.

There are over 100 different types of HPV. Two types have been shown to cause most cases of cervical cancer (around 70% of cases). Vaccination against these two types of HPV is recommended for girls in year 8 at school.

2 Why is this study being done?

Our team is doing research to find out why some girls do get the HPV vaccine and why some girls do not. We would like to hear from parents whose daughters have been offered the HPV vaccine in the past, so we can learn about how they decided whether to let their daughter have the vaccine or not. We would like to speak to parents who are from White British, Black, Asian and minority ethnic communities.

3 Why have I been asked to take part?

All parents of daughters in school years 9 to 11 from local schools have been invited, along with parents from community groups. We are particularly keen to speak to parents of daughters:

- from Black and Asian minority ethnic groups who **did** have the HPV vaccine;
- from Black and Asian minority ethnic groups who **did not** have the HPV vaccine;
- who are from White British backgrounds who **did not** have the HPV vaccine.

4 Do I have to take part?

It is your choice whether you take part or not. Choosing not to take part will not disadvantage you in any way. You can leave the study at any time without having to have a reason.

5 What will happen to me if I agree to take part?

If you agree to take part, please complete the reply slip at the end of this form and send it back to us using the pre-paid envelope. A researcher will contact you to find a good time and place to come and speak to you. The interview will last about an hour. This is all you will be asked to do.

6 What are the possible risks of me taking part?

In the interview you will be asked about your beliefs and previous experiences of vaccinating your daughter. You do not have to answer any question that you do not want to.

7 What are the possible benefits of me taking part?

There are no expected benefits of you taking part. However, the interviews will help us to learn about why some girls might not be having the cervical cancer vaccine. Using this information we can find out about what extra information parents might need to know when they are deciding about whether to let their daughter have the vaccine.

8 Will anyone know that I have agreed to take part?

We will not tell anyone that you have agreed to take part. Only the researchers will have access to your personal information. Recorded interviews will be written up with any identifying information removed. We would like to use the audio recordings when presenting the study findings and for teaching. All information will be collected and stored in accordance with the Data Protection Act 1998.

Other researchers may wish to use the written up interviews for other research. We will only allow this when we are confident that this new research will be done properly.

9 What will happen if I do not want to continue with the study?

You may leave the study at any time without giving a reason.

10 What if there is a problem?

If you have any complaints about the study, you may write to or ask to speak to the researchers who will do their best to answer your questions: Principal Investigator: [name] [telephone number].

11 What will happen to the results of the study?

The results will be published in science journals and presented at national and international meetings. We will use the findings of the study to develop an intervention to give parents the extra information they need to make a decision about whether to let their daughter have the HPV vaccine or not.

12 Who is doing the research and who is paying for it?

The research is being paid for by Cancer Research UK. The project is being done by the [department] at [institution].

13 Contact details

Principal Investigator: [name], [department], [institution], [telephone number].

---✂---

I would like to take part in the EMPATHIC study

My name is: .... My telephone number is: ....

My email address is: .... My address is: ....

....

The best time to contact me is (please circle):

Mon	Tues	Wed	Thu	Fri	Sat	Sun
AM PM	AM PM	AM PM	AM PM	AM PM	AM PM	AM PM

Please detach this slip and return in the pre-paid envelope provided. You do not need a stamp.

The EMPATHIC Study

An invitation to take part in our interview study.

[image]

Summary

Our team is doing research to find out why some girls do get the HPV vaccine and why some girls do not. We would like to speak to parents whose daughters have been offered the HPV vaccine in the past, so we can learn about how they decided whether to let their daughter have the vaccine or not.

We are writing to you to ask if you would take part in this study. If you agree we would come to speak to you about the HPV vaccine. **This would happen at a time and place that is suitable for you.**

The study is being done by researchers from [institution]. Please contact [name] if you would like to talk to her about the study.

Before you decide you need to understand why the study is being done and what it would mean for you. Please read the following information. Talk to your friends and family about the study if you wish.

Please contact us using the details provided at the end of this form if you would like to take part.

[name]

[department], [institution]

[telephone number] [email address]

[institution website address]

Contents

1 What is HPV?
2 Why is this study being done?
3 Why have I been asked to take part?
4 Do I have to take part?
5 What will happen to me if I agree to take part?
6 What are the possible risks of me taking part?
7 What are the possible benefits of me taking part?
8 Will anyone else know that I have agreed to take part?
9 What will happen if I do not want to continue with the study?
10 What if there is a problem?
11 What will happen to the results of the study?
12 Who is doing the research and who is paying for it?
13 Contact details

1 What is HPV?

Human papillomavirus (also known as HPV) affects the skin and moist areas that line the body. It is spread by skin to skin contact. HPV is common and most people will get infected in their life. In most people HPV causes no symptoms and goes away on its own, but for some people the virus can cause cell changes which can increase the risk of some cancers.

There are over 100 different types of HPV. Two types have been shown to cause most cases of cervical cancer (around 70% of cases). Vaccination against these two types of HPV is recommended for girls in year 8 at school.

2 Why is this study being done?

Our team is doing research to find out why some girls do get the HPV vaccine and why some girls do not. We would like to hear from parents whose daughters have been offered the HPV vaccine in the past, so we can learn about how they decided whether to let their daughter have the vaccine or not. We would like to speak to parents who are from White British, Black, Asian and minority ethnic communities.

3 Why have I been asked to take part?

All parents of daughters in school years 9 to 11 from local schools have been invited, along with parents from community groups. We are particularly keen to speak to parents of daughters:

- from Black and Asian minority ethnic groups who **did** have the HPV vaccine;
- from Black and Asian minority ethnic groups who **did not** have the HPV vaccine;
- who are from White British backgrounds who **did not** have the HPV vaccine.

4 Do I have to take part?

It is your choice whether you take part or not. Choosing not to take part will not disadvantage you in any way. You can leave the study at any time without having to have a reason.

5 What will happen to me if I agree to take part?

If you agree to take part, please contact us using the details provided at the end of this form. A researcher will contact you to find a good time and place to come and speak to you. The interview will last about an hour. This is all you will be asked to do.

6 What are the possible risks of me taking part?

In the interview you will be asked about your beliefs and previous experiences of vaccinating your daughter. You do not have to answer any question that you do not want to.

7 What are the possible benefits of me taking part?

There are no expected benefits of you taking part. However, the interviews will help us to learn about why some girls might not be having the cervical cancer vaccine. Using this information we can find out about what extra information parents might need to know when they are deciding about whether to let their daughter have the vaccine.

8 Will anyone know that I have agreed to take part?

We will not tell anyone that you have agreed to take part. Only the researchers will have access to your personal information. Recorded interviews will be written up with any identifying information removed. We would like to use the audio recordings when presenting the study findings and for teaching. All information will be collected and stored in accordance with the Data Protection Act 1998.

Other researchers may wish to use the written up interviews for other research. We will only allow this when we are confident that this new research will be done properly.

9 What will happen if I do not want to continue with the study?

You may leave the study at any time without giving a reason.

10 What if there is a problem?

If you have any complaints about the study, you may write to or ask to speak to the researchers who will do their best to answer your questions: Principal Investigator: [name] [telephone number].

11 What will happen to the results of the study?

The results will be published in science journals and presented at national and international meetings. We will use the findings of the study to develop an intervention to give parents the extra information they need to make a decision about whether to let their daughter have the HPV vaccine or not.

12 Who is doing the research and who is paying for it?

The research is being paid for by Cancer Research UK. The project is being done by the [department] at [institution].

13 Contact details

Principal Investigator: [name], [department], [institution], [telephone number].

If you would like to take part in the study please contact [name] at:
[email address]
[telephone number]

Alternatively, please visit our web page and complete an online form to register your interest: [institution website address]

STUDY ONE

Online recruitment: Facebook advert

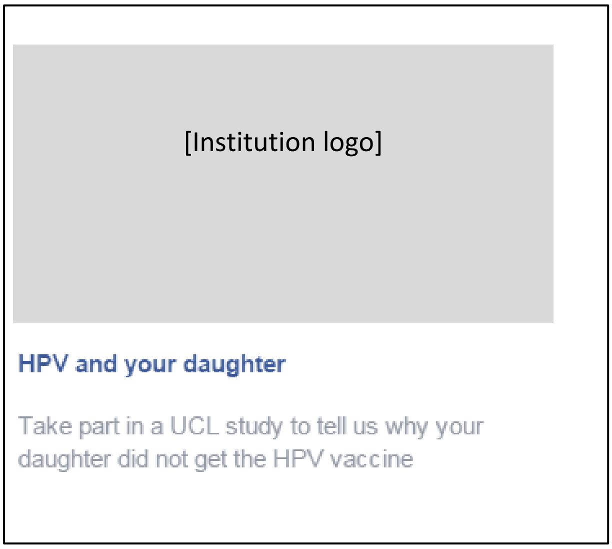

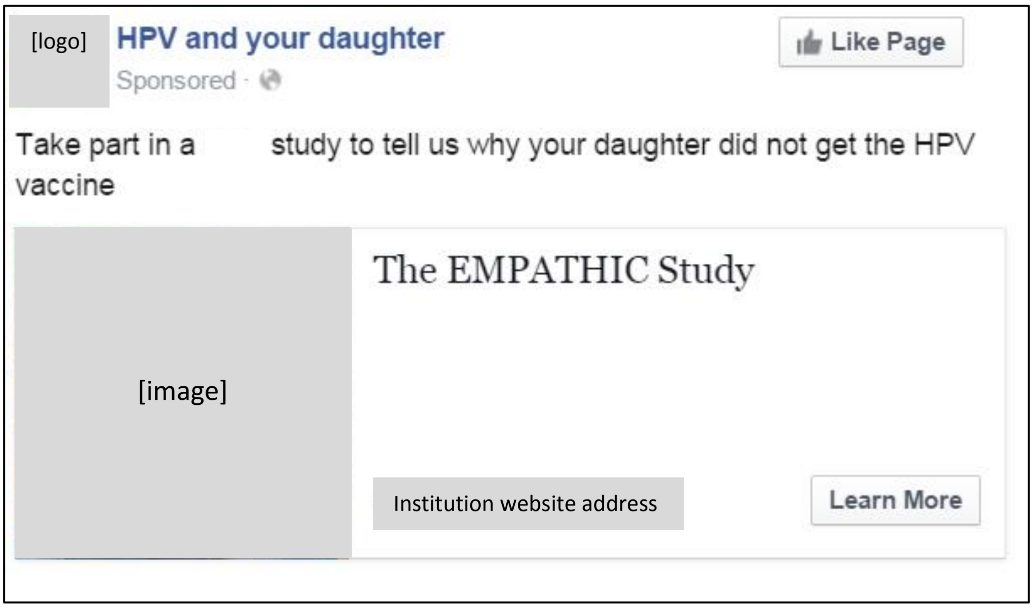

STUDY ONE
Online recruitment: Website posts

[Institution] study: Tell us why your daughter didn't get the HPV vaccination

Our team at [institution] are currently doing research to find out reasons why some girls get the HPV vaccination, whilst some other girls don't. We are looking to interview parents to learn about how they made their decision to let their daughter have the vaccine or not.

We are keen to speak to any Mums (or Dads) who have a daughter between the ages of 13-16, who has not had the HPV vaccination.

Interviews usually take around 30 minutes and can be done in person or over the phone.

If you would like to find out more information about this study please visit our webpage at [institution website address].

If you would like to take part please contact [name] at [email address] or on [telephone number]. Alternatively, please complete the contact form which can be found by clicking here.

We look forward to hearing from you.

[Institution] Study: Your daughter & the HPV vaccination

Our team at [institution] are currently doing research to find out reasons why some girls get the HPV vaccination, whilst some other girls don't. We are looking to interview parents to learn about how they made their decision to let their daughter have the vaccine or not.

We are keen to speak to any Mums (or Dads) who have a daughter between the ages of 13-16, who has not had the HPV vaccination.

Interviews usually take around 30 minutes and can be done in person or over the phone.

If you would like to find out more information about this study please visit our webpage at [institution website address].

If you would like to take part please contact [name] at [email address] or on [telephone number]. Alternatively, please complete the contact form which can be found by clicking on the above link.

We look forward to hearing from you.

[Department]
[Institution]

Funded by CANCER RESEARCH UK

PARTICIPANT INFORMATION SHEET

An interview study about women's views on cervical screening

Summary
We know that about 1 in 4 women in Britain don't take part in cervical screening – either at all, or as often as recommended. This study aims to understand more about why this is, to help make sure we give women the best possible information about screening and make it easy for them to take part if they want to.

We are writing to you to ask if you would like to take part in this study. If you agree, we would like to speak to you about your thoughts and feelings about cervical screening (even if you don't know much about it) and how you think about your health more generally. We will also ask you about different ways of letting women know about screening. **You would talk to a female researcher in person or by phone, at a time and place that is suitable for you.**

The study is being done by researchers from [institution]. Please contact [name] at the [department], [institution] if you would like to talk to her about the study [telephone number].

Please fill in the enclosed slip and send it back in the pre-paid envelope provided if you would like to take part. Please keep this Participant Information Sheet for your reference.

We are writing to you to ask if you would like to take part in a study. In this study we want to understand more about why some women do not go for cervical screening.

Before you decide, you need to know why the study is being done and what it would mean for you. Please read the following information. Talk to your friends and family about the study if you wish.

- Part 1 tells you why the study is being done and what will happen if you agree to take part
- Part 2 gives you more information about how we are going to do the study

Part 1

Why is this study being done?
We know about 1 in 4 women do not go for cervical screening as recommended. Some have never heard of screening. Others have made a clear decision not to go, and some mean to go but haven't yet done so. This study aims to understand more about these different groups. Our findings will help make sure women are given the right information and support to make an informed choice about screening, and can go for screening if they decide they want to.

Why have I been asked to take part?
You recently took part in our survey on cervical screening and women's health as part of a larger TNS survey in January 2016. During that survey, you said you would be interested in taking part in future health-related studies. We are particularly keen to speak to women who:

have never been for screening
no longer go for screening
are not up to date with screening

We believe you fit into one of these groups because of the answers you gave to the survey.

Do I have to take part in the study?
It is your choice whether you take part or not. Choosing not to take part will not disadvantage you in any way. You can leave the study at any time without having to give a reason.

What will happen to me if I agree to take part?
If you agree to take part, please complete the slip that came with this letter and send it back to us using the pre-paid envelope. A researcher will contact you to find a good time (and place, if you choose to be interviewed face-to-face) to speak to you. The interview will last for about an hour. You will be asked to give consent by signing a consent form. This is all you will be asked to do.

What are the possible risks of me taking part?
In the interview you will be asked about your beliefs and previous experiences of cervical screening, as well as other related health topics. You do not have to answer any questions that you do not want to.

What are the possible benefits of me taking part?
There are no expected benefits of you taking part. However, the interviews will help us learn about why some women are not going for cervical screening. Using this information we can find out about what extra information women might need when they are deciding about whether or not to have the screening test.

Will anyone else know that I have agreed to take part?
We will not tell anyone that you have agreed to take part. Only the researchers will have access to your personal information. Recorded interviews will be written up and the recording will then be destroyed. Your name will not be linked to the interview transcript and we will remove any information that might identify you. We will follow ethical and legal practice and all information about you will be private. All information will be collected and stored in accordance with the Data Protection Act 1998.

Other researchers may wish to use the written up interviews for other research. We will only allow this when we are confident that this new research will be done properly. We will never share your personal information (e.g. you name or address) with anyone outside of the research team.

This is the end of Part 1. If the information in Part 1 has interested you and you are thinking about taking part, please read Part 2 before deciding to take part or not.

Part 2

What will happen if I do not want to continue with the study?
You may leave the study at any time without giving a reason.

What if there is a problem?
If you have any complaints about the study, you may write to or ask to speak to the researchers who will do their best to answer your questions: [name]: [telephone number]; [email address].

What will happen to the results of the study?
The results will be published in science journals and presented at national and international meetings. We may use the findings of the study to help develop ways of giving women information about screening, or helping them take part if they want to. You can request a copy of the final report.

Who is doing the research and who is paying for it?
The research is being paid for by Cancer Research UK. The project is being done by the [department].

Contact details
Principal Investigator: [name], [department], [institution], [telephone number], [email address].

STUDY TWO

Online recruitment: Facebook advert

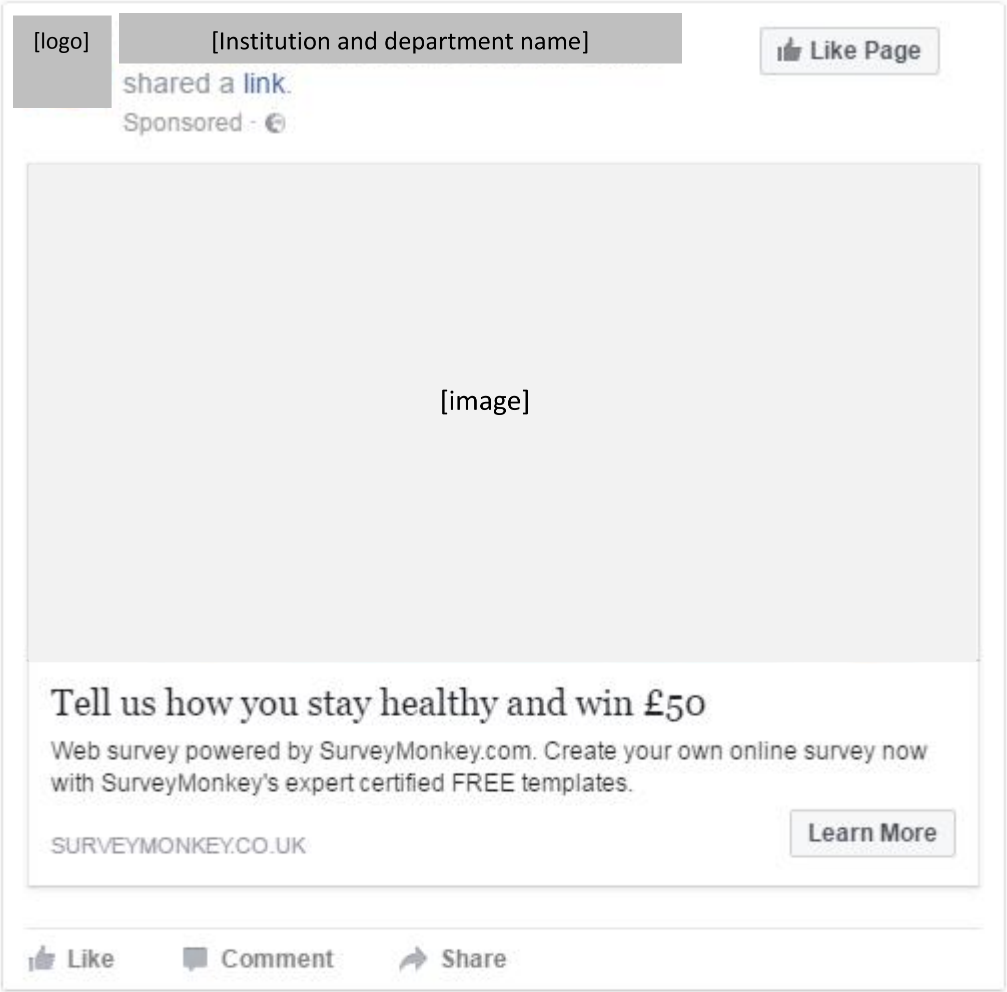

STUDY TWO

Online recruitment: Website posts

[Institute] survey on taking care of your health - chance to win £50 (1 Post)

Wed 23-Nov-16 10:48:46 **Add message** | Report

Hello all,

My name is [name] and I'm a researcher at [institution] [department] We are currently looking into how women aged 25-64 look after their health, and we'd really appreciate people taking our survey - [website address]

By completing the survey you will be entered into a prize draw to win £50 (so long as you provide your email at the end). Depending on your answers you might also be asked if you'd be interested in taking part in an interview study, and if you are interviewed you will be given a £20 Boots voucher as a thank you for your time.

Please feel free to contact me if you have any questions, either on here or by email - [email address]

Thank you so much!
